# Medical Department Harvard University

**Published:** 1872-01

**Authors:** 


MEDICAL DEPARTMENT HARVARD UNIVERSITY.—The number of students in all the departments of Harvard University is not so large as last year by 210 —a falling off of 105 medical students.

The important change in the latter, in the plan of study and the requisites for a medical degree, probably accounts for the deficiency in numbers.—*M. Record.*

